# Supplementary figures and images for: FlgV forms a flagellar motor ring that is required for optimal motility of Helicobacter pylori
Source: PLoS One. 2023 Nov 17;18(11):e0287514. doi: 10.1371/journal.pone.0287514 (PMC10655999; doi:10.1371/journal.pone.0287514)

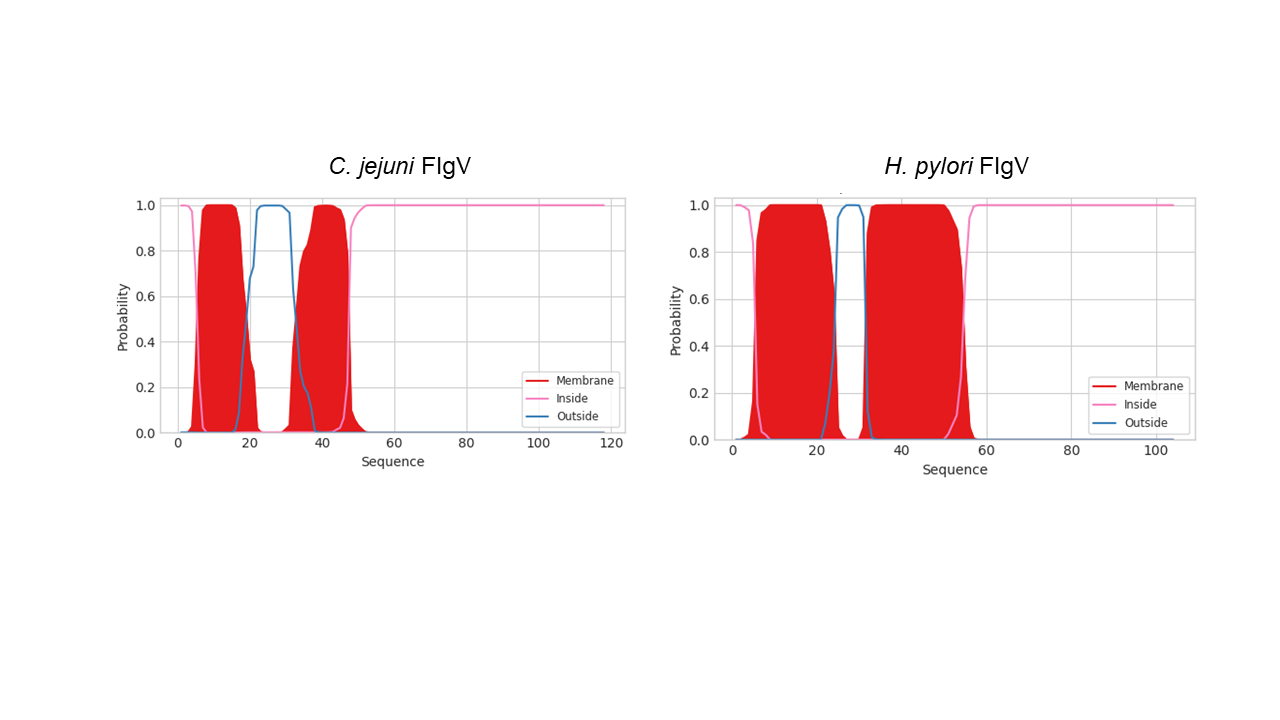

Supplement: S1 Fig — The amino acid sequences of C. jejuni 81–176 and H. pylori G27 FlgV homologs were analyzed using DeepTMHMM (https://dtu.biolib.com/DeepTMHMM) to predict transmembrane topology of the proteins. Predicted transmembrane helices are indicated in orange, regions of the protein predicted to be exposed on the cytoplasmic side of the membrane are indicated by the pink line, and regions of the protein predicted to be exposed on the periplasmic side of the membrane are indicated by the blue line. (TIF) [file pone.0287514.s001.tif]

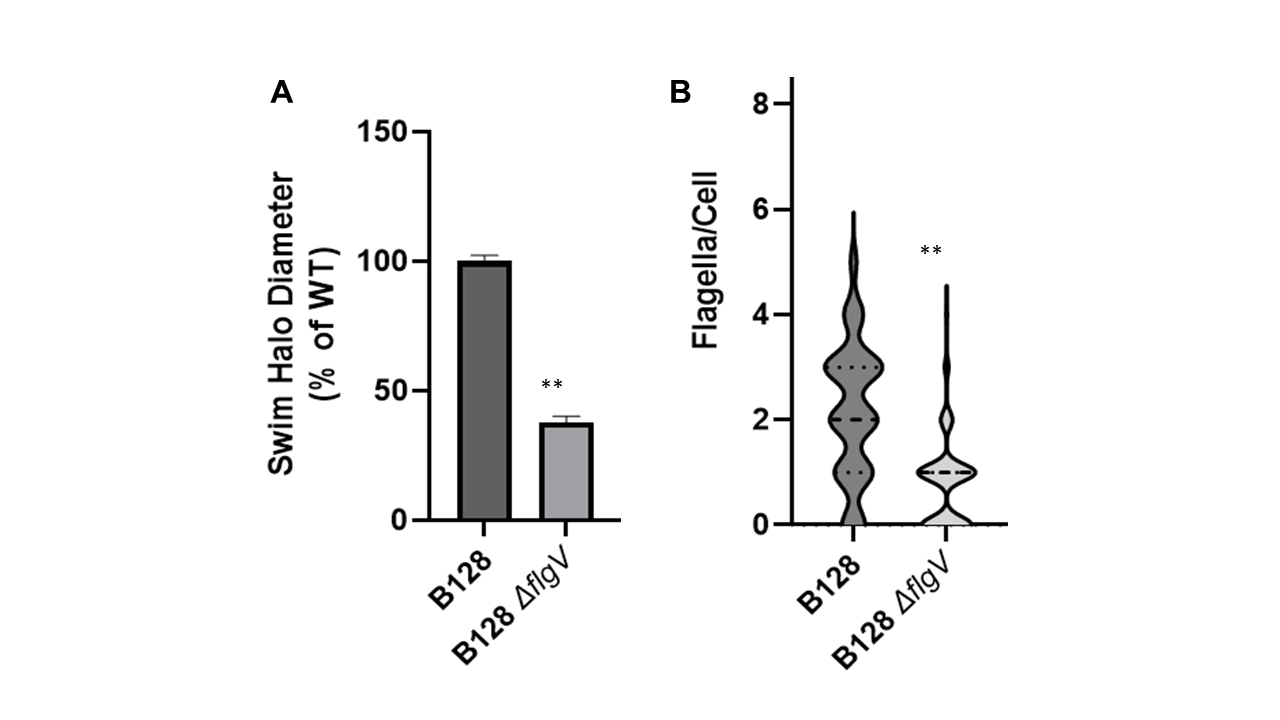

Supplement: S2 Fig — (A) Motilities of H. pylori B128 wild type and H. pylori B128 ΔflgV mutant in soft agar medium. Strains were stab inoculated into soft agar medium, and diameters of the resulting swim halos were measured following 7 d incubation. Bars indicate mean values for swim halo diameters. Three replicates were done for each strain. Error bars indicate standard deviation of the mean. The swim halo diameter of the ΔflgV mutant differed significantly from that of wild type (p-value <0.00001). Statistical analysis of the data was done using a two-sample t test. (B) Flagella were counted for at least 95 cells for H. pylori B128 wild type and H. pylori B128 ΔflgV. Distribution of the number of flagella per cell for ΔflgV mutant differed significantly from that of wild type (p-values <0.00001). Statistical significance for differences in the distribution of the number of flagella per cell were determined using a Mann-Whitney U test. (TIF) [file pone.0287514.s002.tif]

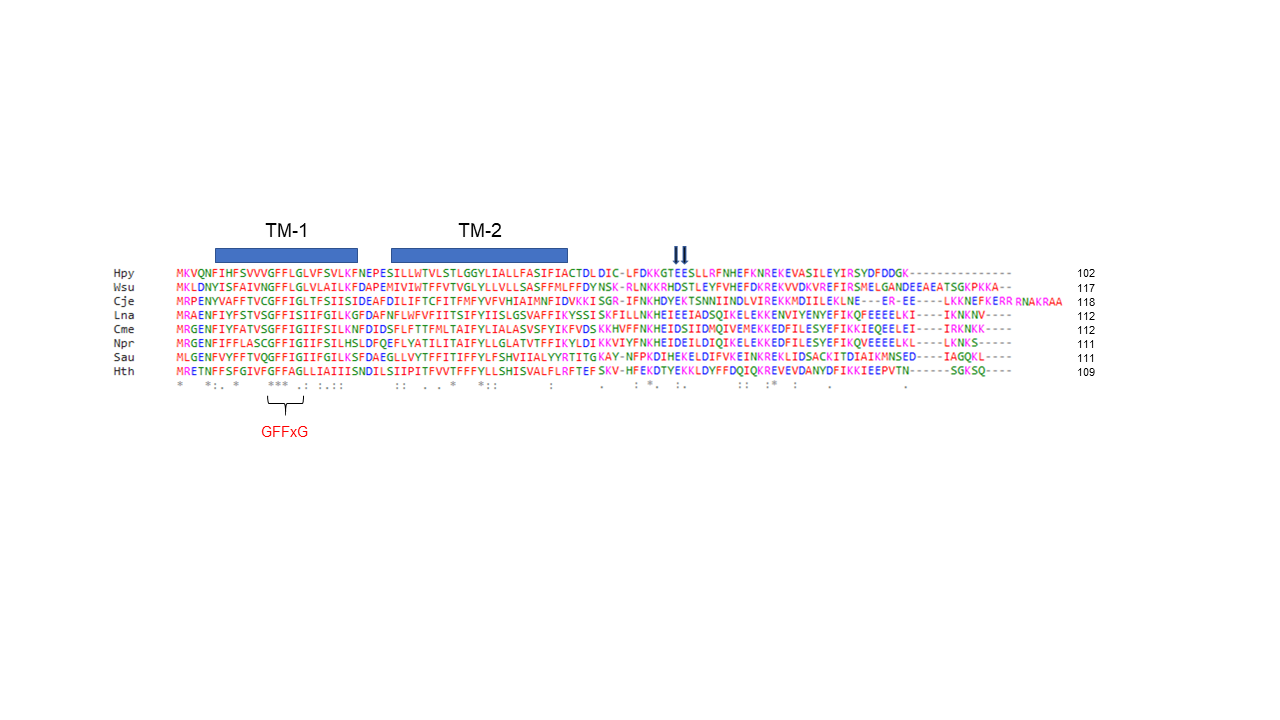

Supplement: S3 Fig — Amino acid sequences of FlgV homologs from H. pylori G27 (Hpy), Wolinella succinogenes DSM 1740 (Wsu), Campylobacter jejuni 81–176 (Cje), Lebetimonas natshushimae (Lna), Caminibacter mediatlanticus (Cme), Nautilia profundicola AM-H (Npr), Sulfurimonas autotrophica DSM 16294 (Sau), and Hydrogenimonas thermophila (Hth) were aligned using Clustal Omega (https://www.ebi.ac.uk/Tools/msa/clustalo/). Transmembrane helices 1 and 2 (TM-1 and TM-2) for H. pylori FlgV are indicated and were predicted using Phobius (https://www.ebi.ac.uk/Tools/pfa/phobius/). The conserved GFFxG motif is indicated. Arrows indicate H. pylori Glu-71 and Glu-72. Numbers indicate the lengths of the FlgV homologs. (TIF) [file pone.0287514.s003.tif]

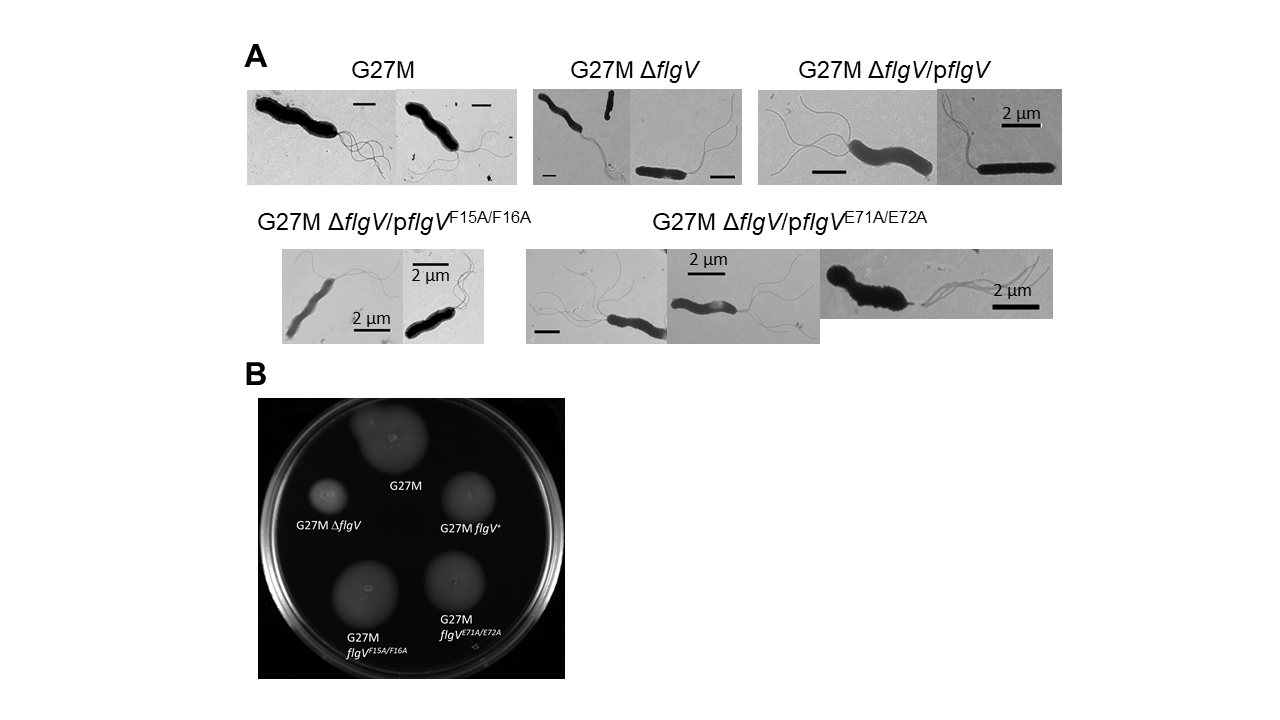

Supplement: S4 Fig — (A) Transmission electron micrographs of representative flagellated cells of H. pylori G27M; H. pylori G27M ΔflgV mutant; and H. pylori G27M ΔflgV mutant bearing the pHel3 vector that expresses wild-type FlgV, FlgVF15A/F16A, or FlgVE71A/E72A. (B) Motility of the H. pylori G27M derived strains in soft agar medium. Photograph of motility plate was taken 7 d post-inoculation. (TIF) [file pone.0287514.s004.tif]

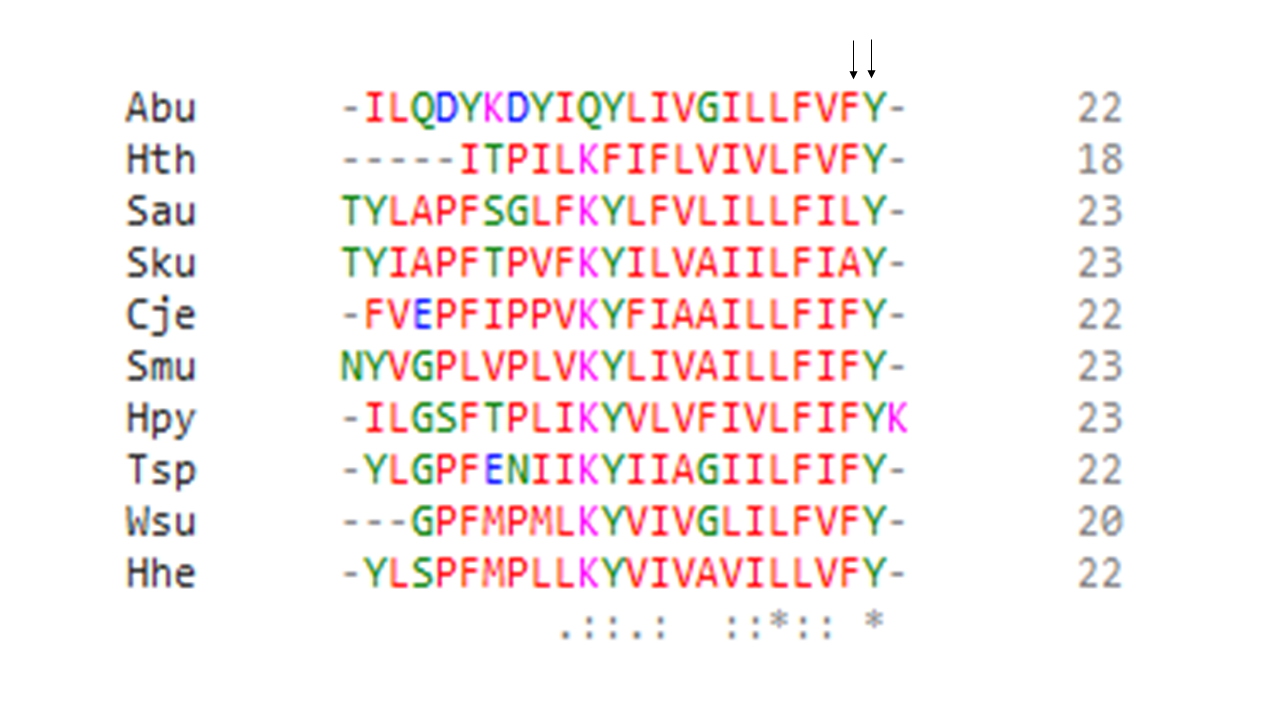

Supplement: S5 Fig — FliF homologs are from Arcobacter butzleri 7h1h (Abu), Hydrogenimonas thermophila (Hth), Sulfurimonas autotrophica DSM 16294 (Sau), Sulfuricurvum kujiense DSM 16994 (Sku), Campylobacter jejuni 81–176 (Cje), Sulfurospirillum multivorans DSM 12446 (Smu), Helicobacter pylori G27 (Hpy), Thiovulum sp. ES (Tsp), Wolinella succinogenes DSM 1740 (Wsu), and Helicobacter hepaticus ATCC 51449 (Hhe). Alignment was done using Clustal Omega (https://www.ebi.ac.uk/Tools/msa/clustalo/). Transmembrane helices were predicted using Phobius (https://www.ebi.ac.uk/Tools/pfa/phobius/). Arrows indicate H. pylori FliF Phe-466 and Tyr-467. Numbers indicate the length of the predicted transmembrane helices. (TIF) [file pone.0287514.s005.tif]

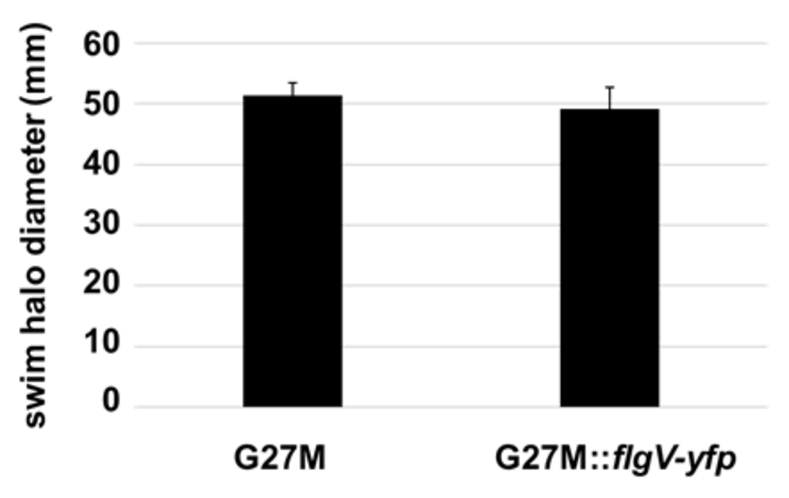

Supplement: S6 Fig — Motilities of H. pylori G27M expressing wild-type FlgV (G27M) or FlgV-YFP fusion protein (G27M::flgV-yfp) in soft agar medium. Strains were stab inoculated into soft agar medium, and diameters of the resulting swim halos were measured following 7-d incubation. Bars indicate mean values for swim halo diameters. Six to eight replicates were done for each strain. Error bars indicate standard deviation of the mean. The mean swim halo diameters of the two strains did not differ significantly from each other (p-value = 0.37). Statistical analysis of the data was done using a two-sample t test. (TIF) [file pone.0287514.s006.tif]
