# Supplementary material for: FlgV forms a flagellar motor ring that is required for optimal motility of Helicobacter pylori
Source: PLoS One. 2023 Nov 17;18(11):e0287514. doi: 10.1371/journal.pone.0287514 (PMC10655999; doi:10.1371/journal.pone.0287514)
Supplement: S1 Table — (DOCX) [file pone.0287514.s007.docx]

**S1 Table. Primers used in this study.**

| **Primer #** | **Name** | **Sequence** |
| --- | --- | --- |
| 74 | flgV R + YFP linker | 5'-CCCGCTCCCAGCGCTCCCTTTTCCATCATCAAAATCATAACT TCTA |
| 75 | YFP F + linker | 5'-GGGAGCGCTGGGAGCGGGATGGTGAGCAAGGGCGA |
| 76 | YFP R + flgV overhang | 5'-TTTTTGATTTCCTTTGGGCATTTTACTTGTACAGCTCGTCCA |
| 77 | flgV DS F + YFP overhang | 5'-TGGACGAGCTGTACAAGTAAAATGCCCAAAGGAATTCAAAAA |
| 78 | flgV DS R | 5'-CAATCGCATTGAATTGCTCAT |
| 85 | flglV US F | 5’-GGGGATAGCGGCGAAGAAATT |
| 86 | flgV US R + overhang | 5’-GCTAGCGATAATCGAATTCCTCGAGAAACCCTACCACAACAG AAAAATG |
| 87 | flgV DS F + overhang | 5’-CTCGAGGAATTCGATTATCGCTAGCAAGAACAGAGAAAAAGA AGTGGCTAGT |
| 88 | flgV DS R | 5’-GTGATTTCATCTTGCTCAATCGCATTG |
| 74 | flgV R + YFP linker | 5'-CCCGCTCCCAGCGCTCCCTTTTCCATCATCAAAATCATAACT TCTA |
| 75 | YFP F + linker | 5'-GGGAGCGCTGGGAGCGGGATGGTGAGCAAGGGCGA |
| 76 | YFP R + flgV overhang | 5'-TTTTTGATTTCCTTTGGGCATTTTACTTGTACAGCTCGTCCA |
| 77 | flgV DS F + YFP overhang | 5'-TGGACGAGCTGTACAAGTAAAATGCCCAAAGGAATTCAAAAA |
| 78 | flgV DS R | 5'-CAATCGCATTGAATTGCTCAT |
| 143 | flhG US F | 5’-ATCACTTTAGACAATTATCGCATTGGG |
| 144 | flhG US R + XhoI + linker + NheI | 5’-CTCGAGGATCGAATCGCTAGCTCCCTTACCGCTTGTGATAGC |
| 145 | flhG DS F + NheI + linker + XhoI | 5’-GCTAGCGATTCGATCCTCGAGTTGAAACGCTATGTGAGGGAG |
| 146 | flhG DS R | 5’-TGGCTAATTTAATCAATTCTTCAGTGC |
| 151 | flgV US F + BspQ1 | 5’-AAGCTCTTCAATGAAAGTGCAAAATTTTATCCATTTTTC |
| 152 | flgV DS R + BspQ1 | 5’-AAGCTCTTCATTATTTTCCATCATCAAAATCATAACTTC |
| 153 | flgV F15A F16A US | 5' CACTAACCCCAAAGCAGCCCCTACCACAAC |
| 154 | flgV F15A F16A DS | 5' TTTTCGGTGTTGAAATTCAATGAGCCAGA |
| 155 | flgV E71A E72A US | 5' TTGTCAAAAAGACAAATATCTAAATCCGTGCAAGCG |
| 156 | flgV E71A E72A DS | 5' AAAAGGCACTGCAGCGAGCTTGCTTCG |
