# Supplementary material for: FlgV forms a flagellar motor ring that is required for optimal motility of Helicobacter pylori
Source: PLoS One. 2023 Nov 17;18(11):e0287514. doi: 10.1371/journal.pone.0287514 (PMC10655999; doi:10.1371/journal.pone.0287514)
Supplement: S2 Table — (DOCX) [file pone.0287514.s008.docx]

**S2 Table. Intragenic mutations identified in *H. pylori* G27M.**

| **Gene** | **Annotation** | ***^a^*Mutation** | ***^b^*Impact** | ***^c^*Freq** |
| --- | --- | --- | --- | --- |
| *HPG27_RS00365 ←* | pseudogene | +A (300/764 nt) |  | 99.20% |
| *HPG27_RS00760 →* | pseudogene | +T (258/581 nt) |  | 99.00% |
| *HPG27_RS00830 ←* | acid‑sensing histidine kinase ArsS | (G)_15-> 16_ (1261/1347 nt) | P421fs | 84.90% |
| *HPG27_RS01025 →* | pseudogene | (G)_6→7_ (41/302 nt) |  | 99.10% |
| *HPG27_RS01365 →* | pseudogene | (T)_6→7_ (79/533 nt) |  | 100.00% |
| *HPG27_RS01370 →* | pseudogene | (A)_5→6_ (372/539 nt) |  | 99.40% |
| *HPG27_RS01610 ←* | pseudogene | (T)_7→6_ (121/241 nt) |  | 99.60% |
| *HPG27_RS01635 →* | flagellar basal body L‑ring protein FlgH | GGG→TGT | G178C | 100.00% |
| *HPG27_RS01635 →* | flagellar basal body L‑ring protein FlgH | GGG→TGT | G178C | 100.00% |
| *HPG27_RS01965 →* | pseudogene | (G)_5→6_ (502/722 nt) |  | 97.00% |
| *HPG27_RS02125 ←* | pseudogene | +T (319/371 nt) |  | 98.60% |
| *HPG27_RS02135 →* | pseudogene | (G)_6→7_ (615/1250 nt) |  | 99.00% |
| *HPG27_RS02165 ←* | pseudogene | (T)_6→7_ (368/539 nt) |  | 99.60% |
| *HPG27_RS02535 ←* | pseudogene | +T (2920/5711 nt) |  | 95.90% |
| *HPG27_RS02655 →* | type IV secretion system oncogenic effector CagA | +T (3637/3693 nt) | S1213fs | 99.50% |
| *HPG27_RS02680 →* | pseudogene | (A)_5→6_ (510/887 nt) |  | 81.10% |
| *HPG27_RS02825 ←* | pseudogene | (T)_6→5_ (487/966 nt) |  | 99.50% |
| *HPG27_RS02825 ←* | pseudogene | (T)_7→6_ (470/966 nt) |  | 100.00% |
| *HPG27_RS02825 ←* | pseudogene | (T)_7→6_ (457/966 nt) |  | 99.40% |
| *HPG27_RS03420 ←* | pseudogene | (A)_7→8_ (1399/2825 nt) |  | 97.90% |
| *HPG27_RS03475 ←* | pseudogene | Δ1 bp (202/1723 nt) |  | 100.00% |
| *HPG27_RS03950 ←* | flagellar basal body‑associated protein FliL | CAG→TAG | Q78* | 100.00% |
| *HPG27_RS04980 ←* | mechanosensitive ion channel family protein | TCT→TTT | S49F | 100.00% |
| *HPG27_RS05755 →* | pseudogene | (A)_5→4_ (2480/3685 nt) |  | 100.00% |
| *HPG27_RS05915 ←* | DUF874 family protein | 2 bp→TG (681‑682/1110 nt) | A228P | 97.70% |
| *HPG27_RS05915 ←* | DUF874 family protein | CAC→CAT | H71H | 100.00% |
| *HPG27_RS05965 ←* | pseudogene | +A (146/8672 nt) |  | 99.60% |
| *HPG27_RS06215 ←* | pseudogene | +T (79/2543 nt) |  | 99.20% |
| *HPG27_RS06225 ←* | pseudogene | C→T (1304/2083 nt) |  | 100.00% |
| *HPG27_RS06225 ←* | pseudogene | A→G (1272/2083 nt) |  | 100.00% |
| *HPG27_RS06225 ←* | pseudogene | C→T (1269/2083 nt) |  | 100.00% |
| *HPG27_RS06225 ←* | pseudogene | C→G (1266/2083 nt) |  | 100.00% |
| *HPG27_RS06225 ←* | pseudogene | T→C (1254/2083 nt) |  | 100.00% |
| *HPG27_RS06225 ←* | pseudogene | C→A (1230/2083 nt) |  | 100.00% |
| *HPG27_RS06225 ←* | pseudogene | A→G (1224/2083 nt) |  | 100.00% |
| *HPG27_RS06225 ←* | pseudogene | G→T (1218/2083 nt) |  | 100.00% |
| *HPG27_RS06225 ←* | pseudogene | T→A (1215/2083 nt) |  | 100.00% |
| *HPG27_RS06225 ←* | pseudogene | 5 bp→5 bp (1208‑1212/2083 nt) |  | 100.00% |
| *HPG27_RS06225 ←* | pseudogene | C→T (1200/2083 nt) |  | 100.0% |
| *HPG27_RS06225 ←* | pseudogene | 2 bp→GC (1197‑1198/2083 nt) |  | 100.00% |
| *HPG27_RS06275 ←* | pseudogene | (A)_8→7_ (1006/1783 nt) |  | 99.10% |
| *HPG27_RS06375 →* | pseudogene | Δ1 bp (384/1542 nt) |  | 100.00% |
| *HPG27_RS06375 →* | pseudogene | Δ1 bp (427/1542 nt) |  | 99.70% |
| *HPG27_RS06375 →* | pseudogene | (T)_6→5_ (491/1542 nt) |  | 100.00% |
| *HPG27_RS06505 ←* | pseudogene | +T (1004/1034 nt) |  | 99.60% |
| *HPG27_RS06835 ←* | pseudogene | (A)_7→6_ (841/886 nt) |  | 99.1% |
| *HPG27_RS08690 →* | pseudogene | T→C (2006/2462 nt) |  | 99.50% |
| *HPG27_RS08690 →* | pseudogene | C→T (2009/2462 nt) |  | 100.00% |
| *HPG27_RS08690 →* | pseudogene | A→G (2036/2462 nt) |  | 100.00% |
| *HPG27_RS08690 →* | pseudogene | 2 bp→GT (2042‑2043/2462 nt) |  | 100.00% |
| *HPG27_RS08690 →* | pseudogene | A→G (2048/2462 nt) |  | 100.00% |
| *HPG27_RS08690 →* | pseudogene | G→A (2051/2462 nt) |  | 99.50% |
| *HPG27_RS08690 →* | pseudogene | G→A (2064/2462 nt) |  | 100.00% |
| *HPG27_RS08690 →* | pseudogene | T→C (2088/2462 nt) |  | 100.00% |
| *HPG27_RS06900 ←* | pseudogene | +A (44/389 nt) |  | 100.00% |
| *HPG27_RS07170 →* | pseudogene | (T)_7→8_ (120/485 nt) |  | 98.80% |
| *HPG27_RS07460 ←* | pseudogene | (A)_5→6_ (1259/2351 nt) |  | 100.00% |
| *HPG27_RS07550 ←* | site‑specific DNA‑methyltransferase | (C)_11→10_ (1368/1386 nt) | E456fs | 91.10% |
| *HPG27_RS07565 →* | pseudogene | (A)_5→6_ (32/635 nt) |  | 98.80% |
| *HPG27_RS07925 ←* | glycosyltransferase family 4 protein | GCG→GTG | A202V | 99.60% |

*^a^*The numbers in parentheses indicate the position of the mutation (first number) within the entire length of the open reading frame (second number).

*^b^*Indicates the position where a different amino acid was introduced, position where a stop codon was introduced (*), or site where a frameshift mutation occurred (fs). No impact is indicated for mutations in pseudogenes.

*^c^*Indicates the percentage of reads at the position that had the particular SNP.
