# Supplementary material for: FlgV forms a flagellar motor ring that is required for optimal motility of Helicobacter pylori
Source: PLoS One. 2023 Nov 17;18(11):e0287514. doi: 10.1371/journal.pone.0287514 (PMC10655999; doi:10.1371/journal.pone.0287514)
Supplement: S3 Table — (DOCX) [file pone.0287514.s009.docx]

**S3 Table. Intergenic mutations identified in *H. pylori* G27M.**

| **^a^Intergenic region** | **Description of flanking genes** | ***^b^*Mutation** | ***^c^*Freq** |
| --- | --- | --- | --- |
| *HPG27_RS00540 ← / ← HPG27_RS00545* | methyl‑accepting chemotaxis protein TlpB/5'‑nucleotidase C‑terminal domain‑containing protein | (C)14→15 (‑76/+132) | 80.00% |
| *HPG27_RS01135 ← / → HPG27_RS01140* | Hop family outer membrane protein HopM/HopN/SulP family inorganic anion transporter | (A)8→9 (‑128/‑113) | 96.90% |
| *HPG27_RS02460 ← / → HPG27_RS02465* | type I glutamate‑‑ammonia ligase/hypothetical protein | (T)6→7 (‑145/‑78) | 98.50% |
| *HPG27_RS02465 → / → HPG27_RS02470* | hypothetical protein/50S ribosomal protein L9 | +C (+313/‑157) | 100.00% |
| *HPG27_RS02745 ← / → HPG27_RS02750* | diaminopimelate epimerase/AI‑2E family transporter | (T)5→6 (‑48/‑68) | 100.00% |
| *HPG27_RS03055 → / → HPG27_RS03060* | sel1 repeat family protein/DUF262 domain‑containing protein | (T)14→13 (+178/‑47) | 89.10% |
| *HPG27_RS04375 ← / ← HPG27_RS04385* | outer membrane beta‑barrel protein/tRNA‑Met | T→C | 99.50% |
| *HPG27_RS04375 ← / ← HPG27_RS04385* | outer membrane beta‑barrel protein/tRNA‑Met | +AAATGCATTTGCATTG | 100% |
| *HPG27_RS04375 ← / ← HPG27_RS04385* | outer membrane beta‑barrel protein/tRNA‑Met | G→A | 99.40% |
| *HPG27_RS04375 ← / ← HPG27_RS04385* | outer membrane beta‑barrel protein/tRNA‑Met | Δ1 bp | 94.00% |
| *HPG27_RS04375 ← / ← HPG27_RS04385* | outer membrane beta‑barrel protein/tRNA‑Met | 4 bp→GGGG | 98.30% |
| *HPG27_RS04475 ← / ← HPG27_RS04480* | outer membrane beta‑barrel protein HofG/TonB‑dependent receptor | (A)15→14 (‑72/+176) | 84.50% |
| *HPG27_RS05845 ← / ← HPG27_RS05850* | NCS2 family permease/Hop family adhesin HopQ | (A)7→6 (‑191/+283) | 100.00% |
| *HPG27_RS05845 ← / ← HPG27_RS05850* | NCS2 family permease/Hop family adhesin HopQ | (T)6→7 (‑410/+64) | 99.10% |
| *HPG27_RS06580 ← / ← HPG27_RS06585* | 50S ribosomal protein L24/50S ribosomal protein L14 | Δ1 bp (‑14/+14) | 99.60% |
| *HPG27_RS06645 → / → HPG27_RS06650* | ATP‑binding protein/hypothetical protein | (TGTATTTA)10→15 (+89/‑153) | 82.8% |
| *HPG27_RS07225 ← / ← HPG27_RS07230* | penicillin‑binding protein activator LpoB/thioredoxin family protein | (A)6→7 (‑315/+102) | 95.00% |
| *HPG27_RS07225 ← / ← HPG27_RS07230* | penicillin‑binding protein activator LpoB/thioredoxin family protein | (A)6→7 (‑331/+86) | 99.40% |
| *HPG27_RS07225 ← / ← HPG27_RS07230* | penicillin‑binding protein activator LpoB/thioredoxin family protein | Δ1 bp (‑350/+67) | 99.50% |
| *HPG27_RS07630 ← / ← HPG27_RS07635* | TonB‑dependent receptor family protein/arginase | (T)18→17 (‑181/+171) | 80.10% |

*^a^*Arrows indicate orientation of genes that flank the intergenic region in which the mutation occurs.

*^b^*The numbers in parentheses indicate the downstream (+) and upstream (-) position of the mutation relative to the flanking genes.

*^d^*Indicates the percentage of reads at the position that had the particular mutation**.**
