# Supplementary material for: FlgV forms a flagellar motor ring that is required for optimal motility of Helicobacter pylori
Source: PLoS One. 2023 Nov 17;18(11):e0287514. doi: 10.1371/journal.pone.0287514 (PMC10655999; doi:10.1371/journal.pone.0287514)
Supplement: Attachment — Submitted filename: Response to reviewers.docx [file pone.0287514.s010.docx]

Eric Cascales
Academic Editor
PLOS ONE

Dear Dr. Cascales,

Thank you for handling the review of the manuscript. We have modified the manuscript based on your comments and the comments of the two reviewers. Below is a point-by-point description of how we addressed the comments, starting with your comments. Our responses follow each specific comment and are in red. We thank both you and the reviewers for the constructive comments as we feel that the suggested changes have significantly improved the manuscript.

Sincerely,

Tim Hoover

Journal Requirements:

When submitting your revision, we need you to address these additional requirements.

1. Please ensure that your manuscript meets PLOS ONE's style requirements, including those for file naming. The PLOS ONE style templates can be found at 
https://journals.plos.org/plosone/s/file?id=wjVg/PLOSOne_formatting_sample_main_body.pdf and 
https://journals.plos.org/plosone/s/file?id=ba62/PLOSOne_formatting_sample_title_authors_affiliations.pdf

We have reformatted the manuscript to meet the PLOS ONE style requirements.

2. Thank you for stating the following in the Acknowledgments Section of your manuscript: 
  "We thank Jennifer Aronson for critical reading of the manuscript. This work was supported by NIH grants AI140444 and AI146907 to T.R.H. and AI087946 and AI132818 to J.L"

We note that you have provided funding information that is not currently declared in your Funding Statement. However, funding information should not appear in the Acknowledgments section or other areas of your manuscript. We will only publish funding information present in the Funding Statement section of the online submission form. 
Please remove any funding-related text from the manuscript and let us know how you would like to update your Funding Statement. Currently, your Funding Statement reads as follows: 
  "This work was supported by National Institutes of Health grants AI140444 (TRH), AI1469077 (TRH), AI087946 (JL), and AI132828 (JI). The URL for NIH is https://www.nih.gov/. The funders had no role in study design, data collection and analysis, decision to publish, or preparation of the manuscript."

Please include your amended statements within your cover letter; we will change the online submission form on your behalf.

The funding statement has been removed from the Acknowledgements Section. The Funding Statement indicated above is correct and does not require any amendment.

3. We note that you have included the phrase “data not shown” in your manuscript. Unfortunately, this does not meet our data sharing requirements. PLOS does not permit references to inaccessible data. We require that authors provide all relevant data within the paper, Supporting Information files, or in an acceptable, public repository. Please add a citation to support this phrase or upload the data that corresponds with these findings to a stable repository (such as Figshare or Dryad) and provide and URLs, DOIs, or accession numbers that may be used to access these data. Or, if the data are not a core part of the research being presented in your study, we ask that you remove the phrase that refers to these data.

We removed the information that was referred to by “data not shown” – see lines 233 and 370 on Revised Manuscript with Track Changes. We feel that deleting these statements has no impact on the data presented in the manuscript or interpretation of the results of the study.

4. Please include a copy of Table 4 which you refer to in your text on page 18.

“Table 4” was a typographical error in the original manuscript. This is Table 1.

5. Please include captions for your Supporting Information files at the end of your manuscript, and update any in-text citations to match accordingly. Please see our Supporting Information guidelines for more information: http://journals.plos.org/plosone/s/supporting-information.

The information has been added.

6. Please review your reference list to ensure that it is complete and correct. If you have cited papers that have been retracted, please include the rationale for doing so in the manuscript text, or remove these references and replace them with relevant current references. Any changes to the reference list should be mentioned in the rebuttal letter that accompanies your revised manuscript. If you need to cite a retracted article, indicate the article’s retracted status in the References list and also include a citation and full reference for the retraction notice.

The references have been checked.

Reviewer #1:
- The title is grammatically poorly formed, and requires an article ("A novel ring of FlgV"?) - although "novel" is over-used and essentially meaningless. How about "A cytoplasmic ring of FlgV is required for optimal..."?

The title has been changed to “FlgV forms a flagellar motor ring that is required for optimal motility of *Helicobacter pylori*”. We have also removed “novel” throughout the manuscript.

- The abstract is misleading, hinting that this is the first time FlgV has been shown to be required for motility: "Here, we provide the first evidence that deletion of flgV in H. pylori B128 and a highly motile variant of H. pylori G27 (G27M) results in reduced motility in soft agar medium.". This fails to capture the fact that FlgV has already been characterised. Gao 2014 identified FlgV using a screen that essentially selected for less motile variants, and as they stated: "Although some mutations could indirectly lead to lack of motility, through a variety of functional, biochemical, and in vivo imaging analyses we have provided strong evidence that the genes we have identified encode factors directly involved in the assembly and/or function of the flagellar apparatus."

We changed the wording of the indicated sentence by replacing “provide the first evidence” to “confirm”. (line 26 in copy with track changes)

- Line 44: MotA has four transmembrane helices, not domains.

The change was made (line 44 of copy with track changes).

- Line 48: It is speculation than MotB rotates within MotA - don't present as fact.

We are not completely clear about the reviewer’s objection, but we modified the sentence to indicate that the MotA pentamer is thought to rotate around the stationary Mot dimer (line 49 of copy with track changes).

- Line 50: "Campylobacterota", not "H. pylori"; worth citing https://mbio.asm.org/content/11/1/e02286-19

We made the change and included the citation (line 51 of copy with track changes).

- Line 91: worth reporting identity/similarity percentages here.

We included the information on identity/similarity (line 226 of copy with track changes).

- Line 99: "strongly suggest" is more appropriate than "strong evidence".

The change was made (line 235 of copy with track changes).

- Line 128: Worth highlighting to non-expert readers that Arcobacter has a mystery, "weird" outlier motor, so this result not expected, but this protein is likely to be FlgV.

We included an additional sentence to indicate that the *Arcobacter* flagellar proteins are divergent to flagellar proteins of other Campylobacterota and included a reference (lines 263-265 of copy with track changes).

- Line 131: I think G27M is a new result? Phrase it as such by writing this sentence in present tense (or provide citation if it's a previous result).

See response to next comment.

- Entire paragraph starting Line 131: I don't understand why you did this work in non-standard strain G27M instead of G27? Was it because you can more sensitively assay motility using G27M? Spell it out to your readers. I'd have preferred you to have mentioned the WT B128 result first, THEN move into a non-standard strain, although don't understand why you used B128 instead of the correct comparison, G27?

We indicate the reason we enriched for a variant of G27 that displayed greater motility in soft agar medium (lines 269-274 of copy with track changes). We do not feel that starting with B128 and then moving to G27M makes as much sense since most of the data presented in the manuscript was obtained with G27M.

- Line 185: Glu 71,72 come out of the blue - need introduction.

We included an introduction for these amino acid residues (line 336 of the copy with track changes).

- Paragraph commencing line 196: this is currently fantasy/speculation. I'd prefer the authors either support this hypothesis with relevant fliF mutant results, or remove or greatly tone-down this paragraph. Use words like "suggestive" and "speculative" to help guide your readers to understand the underlying science.

We disagree that the paragraph needs to be toned down as we feel it is clear that this is a model based on predicted protein-protein interactions. We agree with the reviewer that generating mutations in *fliF* would be a reasonable way to examine the validity of the model, and we hope to do those experiments in the future. To support the confidence of the model, we included a figure showing the predicted aligned error (PAE) score for the model for interactions between FlgV and FliF (Fig 2C), as per Reviewer #2’s recommendation.

- Line 221: worth acknowledging that this density was previously highlighted in Campylobacter jejuni by the yellow arrows in Fig. 2 of http://www.pnas.org/content/early/2016/03/09/1518952113 and that your work is nicely consistent/explanatory with previous work.

We included the suggested statement (lines 372-373 of the copy with track changes).

- Line 237: better phrased, "To further assess whether FlgV forms..." (in science we don't try to obtain evidence for a specific hypothesis, as this biases results - rather, we put hypotheses to the test).

The sentence was modified (line 392 of the copy with track changes).

- Figure 4: labels need to be larger.

The labels on Figure 4 have been enlarged.

Reviewer #2:

- Lines 64-66: Some of the mentioned symmetries were shown to be artefacts of FliF in isolation, and were not not found in FliF within the full flagellum HBB structure (Tan et al, Cell, 2021). The true oligomeric state of FliF is 34, with the inner ring (RBM2) having 23-fold symmetry.

We made the changes in the Introduction (lines 66-68 of the copy with track changes) and Discussion (lines 471 and 475 of the copy with track changes).

- Figure 1b: What does the last operon correspond to?

The figure has been fixed.

- Lines 96-97: I think it would be relevant to show the predicted TM topology, in the supplementary material.

A figure showing the predicted membrane topology has been included (S1 Fig).

- Figure 2C: For AlphaFold modelling, it's essential to show the pLDDT plot, and/or the model Color-coded by confidence, to get a sense of how accurate the prediction is.

A figure showing the PAE score has been added (Fig 2C). The original Figure 2C is not Figure 2D.

- Figure 2: For completeness, a photo of agar plate, a selection of electron micrograph of cells used for counting the flagella, and a table including the number of flagella for each mutant, should be added in the supplementary material.

A figure showing representative electron micrographs of flagellated cells of G27M and G27M-derived strains, together with a photo of a plate for the soft agar motility assays has been added (S4 Fig).

- Lines 179-183: Showing a multiple alignment of the FlgV sequences, with the GxxxG motif indicated and the predicted tomopogy, would be really helpful.

A figure showing multiple alignments of FlgV homologs has been included (S3 Fig).

- Figure 3I, J: These would gain to have additional labels for the FliL ring, which is only mentioned in the legend.

The FliL-rings are indicated in the images.

- Figure 3: Could the authors do a map subtraction, between WT and DflgV, and also between WT and flgV-YFP, to better highlight the regions of density that have changed?

We used "map subtraction" between WT and the mutant to better highlight the FlgV ring as shown in the NEW Figure 4E, F. The difference between WT and flgV-YFP could be better visualized in Fig. 3D.

- Line 250: This could gain from additional labels, an/or a self-rotation analysis to better illustrate the proposed stoichiometry.

We included additional labels. The symmetries of the C-ring and the FlgV-ring are 39 and 21, respectively.

- Figure 4: The model suggests that FlgV is in partly in the IM - should be shown in 4G. Does the non-TM region of the protein fits in the density?

FlgV is inserted in the curved inner membrane. We also resolved 21-fold symmetry of the FlgV ring. However, we do not know how many subunits of FlgV are required for the formation of the FlgV ring or if there are other proteins associated with the density that we attribute to FlgV. Therefore, we do not feel convinced to fit the FlgV model into the density.
